# A Rare Arterial Variant Combining Hepatosplenic and Gastromesenteric Trunks With Bilateral Accessory Renal Arteries

**DOI:** 10.7759/cureus.91801

**Published:** 2025-09-07

**Authors:** George Triantafyllou, Nikolaos-Achilleas Arkoudis, Alexandros Samolis, Georgios Velonakis, Maria Piagkou

**Affiliations:** 1 Department of Anatomy, School of Medicine, Faculty of Health Sciences, National and Kapodistrian University of Athens, Athens, GRC; 2 Second Department of Radiology, School of Medicine, “Attikon” University General Hospital, National and Kapodistrian University of Athens, Athens, GRC

**Keywords:** accessory renal artery, coeliac trunk, gastromesenteric trunk, hepatosplenic trunk, vascular variation

## Abstract

This study details an extremely rare vascular anomaly found incidentally in a 78-year-old male during computed tomography angiography (CTA). The patient had a hepatosplenic trunk, where the common hepatic and splenic arteries arose from the coeliac trunk, and the left gastric artery originated abnormally from the superior mesenteric artery, forming a gastromesenteric trunk. Additionally, bilateral accessory renal arteries were present. While accessory renal arteries are fairly common, the combination of hepatosplenic and gastromesenteric trunks is very rare, with only a few cases documented in the literature, and even fewer with detailed imaging. These arterial variations, though often asymptomatic, have significant implications for hepatopancreatobiliary surgery, interventional radiology, and renal transplantation. Celiac trunk anomalies can complicate dissection or embolization procedures, and accessory renal arteries increase the risk of ureteral injury, make transplantation more technically challenging, and can lead to delayed graft function and decreased graft survival. This case highlights the importance of high-resolution CTA with 3D reconstruction for preoperative vascular mapping to improve surgical planning and reduce intraoperative risks.

## Introduction

The abdominal aorta (AA) supplies the abdominal organs through several major branches, including the coeliac trunk (CeT), the superior and inferior mesenteric arteries (SMA and IMA). Typically, the CeT splits into the left gastric artery (LGA), common hepatic artery (CHA), and splenic artery (SA). At the same time, the SMA and IMA supply the midgut and hindgut structures, respectively. Additional paired arteries, such as the renal arteries (RAs), serve the kidneys and show notable anatomical variation [1].

Morphological variations of these vessels have been extensively studied, with recent high-quality meta-analyses providing consolidated data on their prevalence and clinical relevalence [2-4]. Common variants include CeT bifurcations - most often hepatosplenic (HST) or gastrosplenic (GST) trunks - and accessory RAs, with pooled prevalences of 10.53% and 21.10%, respectively [2,4].

This study aimed to document and illustrate an exceptionally rare arterial constellation, using high-resolution computed tomography angiography (CTA). It underscores the critical importance of high-resolution computed tomography angiography (CTA) in detecting rare deviations that may influence surgical planning, particularly in hepatopancreatobiliary, renal, and transplant procedures.

## Case presentation

During a CTA assessment of a 78-year-old male patient, an unusual arterial configuration was identified and examined in detail due to its rare morphology. The scan, obtained from “Attikon” University Hospital following informed consent, was evaluated using Horos software version 3.3.6 (New York City, NY: Horos Project). Multiplanar reconstructions (axial, coronal, and sagittal) and three-dimensional volume-rendered images were used to assess the vascular anatomy (slice thickness: 1.0 mm).

**Figure 1:**
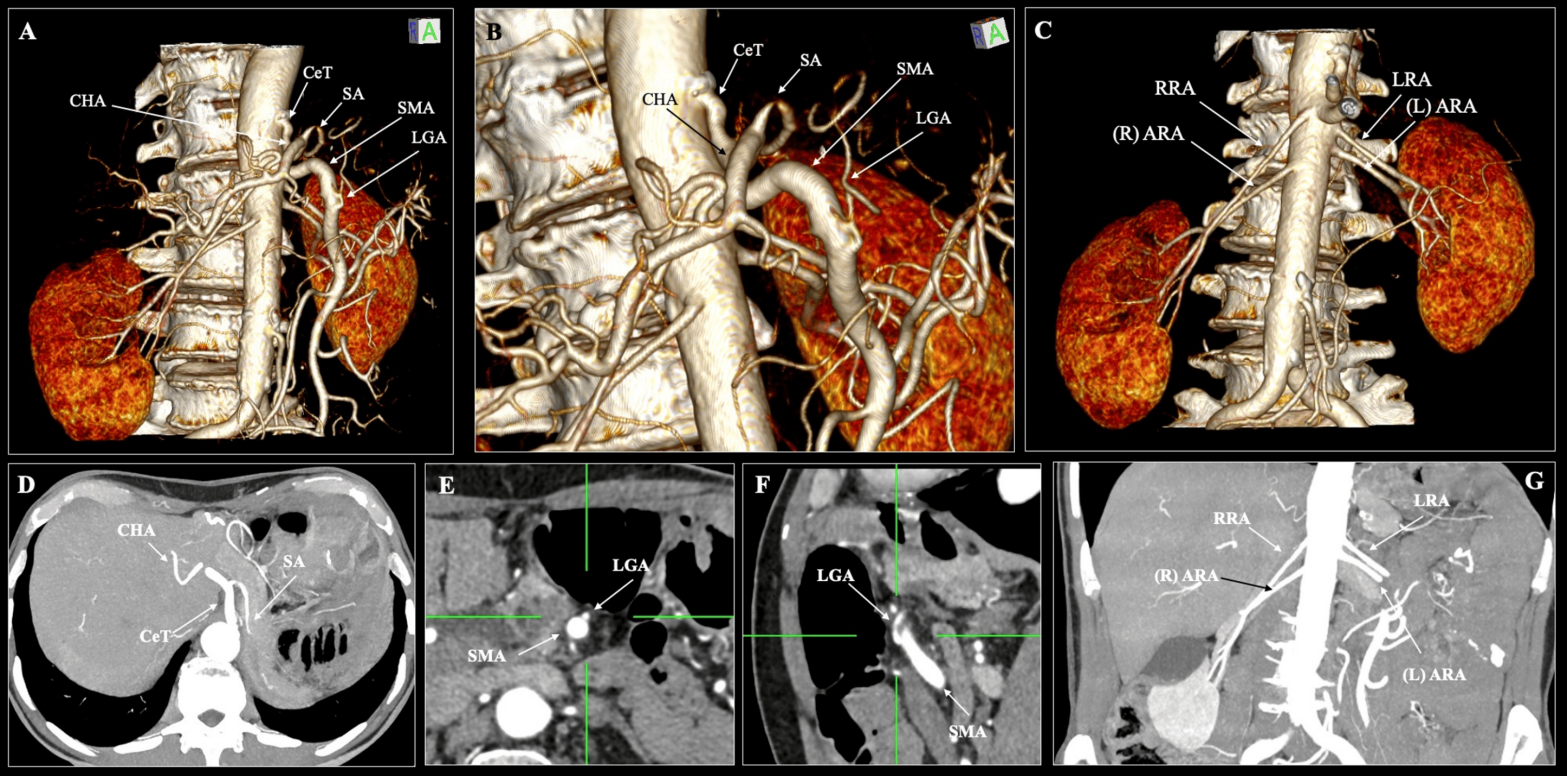
Computed tomography angiography (CTA) imaging of rare abdominal arterial variants. (A, B) Three-dimensional (3D) volume-rendered reconstructions demonstrate the co-occurrence of a hepatosplenic trunk (HST) and a gastromesenteric trunk (GMT). The coeliac trunk (CeT) bifurcates into the common hepatic artery (CHA) and splenic artery (SA). In contrast, the left gastric artery (LGA) arises anomalously from the superior mesenteric artery (SMA) forming a GMT. (C) 3D volume-rendered reconstructions demonstrate the bilateral accessory renal arteries (ARA). (D) Maximum intensity projection (MIP) of axial slice depicting the CeT bifurcating into the CHA and SA. (E, F) Multiplanar reconstruction (MPR) of axial and sagittal slices demonstrating the LGA origin from the SMA. (G) MIP of coronal slice depicting the bilateral ARA. CeT: coeliac trunk; CHA: common hepatic artery; SA: splenic artery; LGA: left gastric artery; SMA: superior mesenteric artery; RRA: right renal artery; LRA: left renal artery; ARA: accessory renal artery.

The CeT originated as the first anterior branch of the AA, with a diameter of 2.97 mm. After a short course of 8.24 mm, the CeT bifurcated into the CHA and the SA, forming a classic HST. The branching pattern of both arteries was typical (Figures 1A, 1B). Notably, the LGA did not arise from the CeT but instead originated as the first branch of the SMA, which itself arose 2.85 mm distal to the CeT and measured 7.87 mm in diameter. The LGA was located 36.7 mm distally to the aortic origin of the SMA, forming a GMT (Figure 1A-1G). Therefore, we observed a dual-trunk pattern, combining an HST and GMT. Further distally, an additional vascular anomaly was noted. At a distance of 8.08 mm below the SMA origin, the AA gave rise to two RAs per kidney. On the left side, the primary RA measured 3.26 mm in diameter, with an accessory RA (ARA) 1.57 mm inferior to it, measuring 3.07 mm. On the right, the main RA measured 5.06 mm, and a second RA arose at 15.3 mm caudal to it, measuring 4.85 mm (Figures 1F, 1G).

## Discussion

Embryologically, such variants arise from the complex development of the ventral splanchnic arteries. The CeT and SMA originate from primitive ventral segmental branches of the aorta, which are initially interconnected by longitudinal anastomoses. Aberrant persistence or regression of these channels explains deviations from the classical trifurcation, including HST or GMT [5,6]. Likewise, ARA results from the persistence of more than one mesonephric artery, a common developmental feature given their role in successive embryonic kidneys [4]. Thus, the coexistence of these anomalies in a single individual reflects parallel embryological processes of vascular remodeling.

The morphological variability of the CeT has been extensively documented in anatomical and radiological literature. Our recent meta-analysis systematically classified these variants based on the number and origin of branching arteries, consolidating data from over 34,000 patients [2]. While the most common variation involves a bifurcation of the CeT, its anatomical combinations vary widely. In the present case, we identified an HST, in which the CHA and SA arise from the CeT, while the LGA atypically originated from the SMA. This configuration defines a GMT and represents an exceptionally rare arterial variant [2].

In our previously mentioned meta-analysis, this specific variant was identified in only three out of 34,095 patients, highlighting its extreme rarity [2]. Among studies, the study by Song et al. was the first to document this variation in a large radiological cohort of 5,002 individuals (0.02%) [6]. Later, Thangarajah and Parthasarathy and Hafezji and Gupta reported similar findings in isolated cases; however, neither study provided detailed imaging evidence [7,8]. Therefore, this report is likely the fourth documented case and only the second to include high-resolution CTA with photographic documentation, further emphasizing its clinical importance.

From a surgical perspective, variants like the LGA originating from the SMA, deviating from its usual origin at the CeT, pose significant risks during upper abdominal procedures, including hepatopancreatobiliary surgery, oncologic resections, and transplantations [5,6]. Unrecognized anomalies can lead to accidental vascular injury, ischemia, or failed embolization during interventional radiology [5,6]. Preoperative identification using multiplanar reconstruction and 3D CTA, as demonstrated in this case, enables personalized surgical plans and reduces intraoperative complications [5,6].

In addition to the rare visceral trunk pattern, this patient exhibited bilateral ARAs, a relatively common variant with a pooled prevalence of 21.10% based on over 54,000 kidneys [4]. Specifically, bilateral single ARAs occur in approximately 5.15% of the population [4]. While frequent in isolation, their coexistence with such an unusual visceral arterial configuration creates a unique formation of the arterial network [4].

Although ARAs are often asymptomatic and found incidentally, their presence significantly impacts urological and transplant surgery. A comprehensive meta-analysis by Zorgdrager et al. reported that patients with ARAs experienced higher rates of surgical complications, delayed graft function, and lower one-year graft survival [9]. Similarly, Afriansyah et al. found that although donor outcomes were similar in laparoscopic nephrectomies, recipient outcomes were negatively affected by the presence of ARAs, requiring more complex vascular reconstruction or multiple anastomoses [10].

## Conclusions

To conclude, the occurrence of trunk variants (HST and GMT) along with bilateral ARAs in a single patient constitutes a rare anatomical variation. This case represents a unique arterial constellation, expanding the known spectrum of vascular variations in the abdomen and emphasizing the importance of thorough vascular mapping preoperatively.
